# Supplementary material for: Virtual Screening of Alkaloid and Terpenoid Inhibitors of SMT Expressed in Naegleria sp
Source: Molecules. 2022 Sep 5;27(17):5727. doi: 10.3390/molecules27175727 (PMC9457665; doi:10.3390/molecules27175727)
Supplement: Supplementary file 1 [file molecules-27-05727-s001.zip › molecules-1817698-supplementary.pdf]

Table S1. Amino acid sequence comparison and percentage identity using ClustalW, Emboss Needle alignment for Yeast, XM, XP. Alignment studies and amino acid comparison for Three protein sequences and the Swiss-model sequences.

YEAST vs XM complete protein vs XP complete protein	Percent Identity Matrix - created by Clustal2.1

```
  1: YEAST         100.00   40.69   41.33
  2: XM_COMPLETE    40.69  100.00   77.56
  3: XP_COMPLETE    41.33   77.56  100.00

YEAST           ----------MSETELRKRQAQFTRELHGDDIGKKTGLSALMSKNNSAQKEAVQKYLRNW
XM_COMPLETE     MSLFLLGVVSIVVIGFVVYLFKFKNQIKGYHL------------TNENVTGTYQDLF---
XP_COMPLETE     --MFLI-ILTVIVGGFILYLFKFREQIKGSHL------------TDTDKTASYQELF---
                          :    :     :*  :::*  :            .:   . : *. :

YEAST           DGRTDKDAEERRLEDYNEATHSYYNVVTDFYEYGWGSSFHFSRFYKGESFAASIARHEHY
XM_COMPLETE     -AQDTKDTHDKRKNAGWDVAGKYYDMVTDFYLYGWGRSFHFAPRHKKESMIESIQRHEYW
XP_COMPLETE     -ATDNQQTHEKRKKAGWDVAGKYYDMVTDFYLYGWGRSFHFATRHSRESLIESILRHEYW
                 .   ::: :.* :  .:.: .**::***** **** ****:  :. **:  ** ***::

YEAST           LAYKAGIQRGDLVLDVGCGVGGPAREIARFTGCNVIGLNNNDYQIAKAKYYAKKYNLSDQ
XM_COMPLETE     LAKQMDLKKGMKCLDLGCGVMGPATNISRFTGAHITGVNNHPYQSQQAKEYISQMGLSEQ
XP_COMPLETE     LAKQLDLKPGMKCLDLGCGVMGPAVNIARFSGCNVTGVNNHPYQSERAKVFINEMGMDGR
                ** : .:: *   **:**** *** :*:**:*.:: *:**: **  .** : .: .:. .

YEAST           MDFVKGDFMKM----DFEENTFDKVYAIEATCHAPKLEGVYSEIYKVLKPGGTFAVYEWV
XM_COMPLETE     CQIVRGDFNNLDDNSDLPSESYDAAYTIEASCHAKDRPHCYKQIYNKLKPGAIFAGYEWV
XP_COMPLETE     CNIVRGDFNNLDDNKDLPAESYDAAYAIEATCHAKDRPHCYKQIFNKLKPGAVFGGYEWV
                 ::*.*** ::    *:  :::* .*:***:*** .    *.:*:: ****. *. ****

YEAST           M-TDKYDENNPEHRKIAYEIELGDGIPKMFHVDVARKALKNCGFEVLVSEDLADND--DE
XM_COMPLETE     MISGKYDSKNEEHNKIKFDIMKGDGLPEILMDKEIDESLRKAGFEVIKTEDVGVTDQIHP
XP_COMPLETE     MITGKYDSKNEEHNKIKFDIMKGDGLPEILMDKEIDDALVKAGFEVIRTEDVAITDKINP
                * :.***.:* **.** ::*  ***:*:::  .   .:* :.****: :**:. .*

YEAST           IPWYYPL-TGEWKYVQNLANLATFFRTSYLGRQFTTAMVTVMEKLGLAPEGSKEVTAALE
XM_COMPLETE     VPWYQPIDNGGW-------DFTSWFQTSY-GRFIVHKLVGILESVGLVPKSSQQAYEFLM
XP_COMPLETE     IPWYQPLDNGGW-------ELTNWFQTSY-GRWVVHKLVGILEKIGLVPKTSQQAYEFLM
                :*** *: .* *       ::.:*.***  ** ..  :* ::*.:**.*: *::.   *

YEAST           NAAVGLVAGGKSKLFTPMMLFVARKPENAETPSQTSQEATQ
XM_COMPLETE     AGASGLVAGGKTGIFTPCYFFMARKPLTAAE----------
XP_COMPLETE     AGA-GLVGGGKTGIFTPSYFFLARKPLKN------------
                 .* ***.***: :***  :*:**** .
```

Yeast vs XM Swiss-model vs XP Swiss-model

Percent Identity Matrix - created by Clustal2.1

1:	YEAST	100.00	44.03	45.78
2:	XM_SWISS	44.03	100.00	80.88
3:	XP_SWISS	45.78	80.88	100.00

```
YEAST           MSETELRKRQAQFTRELHGDDIGKKTGLSALMSKNNSAQKEAVQKYLRNWDGRTDKDAEE
XM_SWISS        ------------------------------------------------------------
XP_SWISS        ------------------------------------------------------------


YEAST           RRLEDYNEATHSYYNVVTDFYEYGWGSSFHFSRFYKGESFAASIARHEHYLAYKAGIQRG
XM_SWISS        -------------------------------------------------------DLKKG
XP_SWISS        ----------------------------------------------------KQLDLKPG
                                                                       .:: *

YEAST           DLVLDVGCGVGGPAREIARFTGCNVIGLNNNDYQIAKAKYYAKKYNLSDQMDFVKGDFMK
XM_SWISS        MKCLDLGCGVMGPATNISRFTGAHITGVNNHPYQSQQAKEYISQMGLSEQCQIVRGDFNN
XP_SWISS        MKCLDLGCGVMGPAVNIARFSGCNVTGVNNHPYQSERAKVFINEMGMDGRCNIVRGDFNN
                   **:**** *** :*:**:*.:: *:**: **  .** : .: .:. . ::*.*** :

YEAST           M----DFEENTFDKVYAIEATCHAPKLEGVYSEIYKVLKPGGTFAVYEWVM-TDKYDENN
XM_SWISS        LDDNSDLPSESYDAAYTIEASCHAKDRPHCYKQIYNKLKPGAIFAGYEWVMISGKYDSKN
XP_SWISS        LDDNKDLPAESYDAAYAIEATCHAKDRPHCYKQIFNKLKPGAVFGGYEWVMITGKYDSKN
                :    *:  :::* .*:***:*** .    *.:*:: ****. *. ***** :.***.:*

YEAST           PEHRKIAYEIELGDGIPKMFHVDVARKALKNCGFEVLVSEDLADND--DEIPWYYPL-TG
XM_SWISS        EEHNKIKFDIMKGDGLPEILMDKEIDESLRKAGFEVIKTEDVGVTDQIHPVPWYQPIDNG
XP_SWISS        EEHNKIKFDIMKGDGLPEILMDKEIDDALVKAGFEVIRTEDVAITDKINPIPWYQPLDNG
                 **.** ::*  ***:*:::  .   .:* :.****: :**:. .*    :*** *: .*

YEAST           EWKYVQNLANLATFFRTSYLGRQFTTAMVTVMEKLGLAPEGSKEVTAALENAAVGLVAGG
XM_SWISS        GW-------DFTSWFQTSY-GRFIVHKLVGILESVGLVPKSSQQAYEFLMAGASGLVAGG
XP_SWISS        GW-------ELTNWFQTSY-GRWVVHKLVGILEKIGLVPKTSQQAYEFLMAGAEGLVGGG
                 *       :::.:*.*** ** ..  :* ::*.:**.*: *::.   *  .* ***.**

YEAST           KSKLFTPMMLFVARKPENAETPSQTSQEATQ
XM_SWISS        KTGIFTPCYFFMAR-----------------
XP_SWISS        KTGIFTPSYFFLARKPL--------------
                *: :***  :*:**
```

Table S2. Yeast Top ligand Binding pockets and their descriptions.

Pocket	Volume [Å³]	Drug Score	Hydrogen bond donors	Hydrogen bond acceptors	Hydrophobic interactions	Negative amino acids	Positive Amino acids
Orange	1869.89	0.81	31	126	19	8	7
Purple	425.73	0.6	15	38	21	5	4
Green	250.82	0.46	8	16	16	2	2

Table S3. Pockets for yeast SMT and the amino acids involved.

Pocket	Amino acids involved
Orange	GLU3, LEU6, ARG7, GLN10, GLY27, LEU28, LEU31, MET32, GLN39, TYR66, ASN67,THR70, HIS71, TYR73, TYR74, VAL77, ASP79, PHE80, TYR83, TRP85, SER87, PHE89, HIS90, PHE91, SER92, PHE94, PHE100, ILE104, GLU 108, ASP125, VAL126, GLY127, CYS128, GLY129, VAL130, GLY131, GLY132, PRO133, LEU148, ASN149, ASN150, ASN151, GLN154, LYS175, GLY176, ASP177, PHE178, TYR192, ALA193, ILE194, GLU195, ALA196, CYS198, HIS199, ALA200, TRP225, GLY248, ASP249, ILE251, TRP286, PRO289, LEU290, ALA304, PHE307, ARG308, THR309, GLY313, ARG314, PHE 316, THR317, MET320, VAL321, MET324, ALA330, PRO331, SER334, VAL 337, THR338, LEU341, GLU342, ALA325, LEU348
Purple	SER92, ARG93, PHE94, VAL226, THR228, ASP229, LYS230, VAL257, ARG261, ASP276, LEU277, ALA278, ASP279, ASN280, ASP281, ASP281, ILE284, PRO285, TRP286, TYR287, GLY351, GLY352, LYS355, LEU356, PHE357, THR358, MET360, MET361
Green	TRP85, GLY86, SER87, SER88, PHE100, ALA101, ILE104, ALA105, GLU108, GLY132, ARG135, GLU136, TYR165, LEU165

Table S4. XP Top ligand binding pockets and their descriptions

Pocket	Volume [Å³]	Drug Score	Hydrogen bond donors	Hydrogen bond acceptors	Hydrophobic interactions	Negative amino acids	Positive Amino acids
Orange	576.58	0.86	13	46	38	4	5
Purple	516.35	0.75	9	45	38	2	2
Green	113.98	0.18	4	16	8	2	1

Table S5. Pockets for XP SMT and the amino acids involved.

Pocket	Amino acids involved
Orange	ASP116, LEU117, GLY118, CYS 119, GLY120, PRO124, VAL139, ASN140, HIS142, GLN145, GLY149, ASP168, PHE169, ASN170, ASN171, LYS73176, ALA188, ILE189, GLU190, ALA191, THR192, HIS194, ALA195, ASP197, HIS200, CYS201, GLN204, LYS208
Purple	TRP220, VAL221, MET222, ILE223, ASP228, SER229, LYS230, ASN231, GLU233, HIS234, ASN235, ILE241, MET242, ILE250, TYR 285, GLN286, LEU339, GLY341, GLY342, GLY343, THR345, GLY356, PRO350, SER351
Green	ASN235, ASP240, MET242, LYS243, LEU247, PRO248, GLU249, ILE250

Table S6. XM Top ligand binding pockets and their descriptions.

Pocket	Volume [Å³]	Drug Score	Hydrogen bond donors	Hydrogen bond acceptors	Hydrophobic interactions	Negative amino acids	Positive Amino acids
Orange	1011.26	0.81	23	59	60	4	4
Purple	577.86	0.78	11	42	42	5	5
Green	212.42	0.41	3	16	13	3	1

Table S7. Pockets for XM SMT and the amino acids involved.

Pocket	Amino acids involved
Orange	ASP110, LEU111, CYS117, PRO127, ASN130, ILE131, PHE134, THR135, ALA188, AA189, TRY190, ILE192, GLU193, ILE217, PHE218, ALA219, TRY221, GLU222, TRP223, MET225, GLY228, LYS229, TYR230, HIS237, LYS241, GLY277, VAL278, THR279, ASP280, GLN281, PRO284, VAL285, PRO286, TRP287, SER305, PHE309, ILE310, LYS313, LEU314, ILE317, LEU318, LEU342, GLY349, ILE350, PHE351, THR352, PRO353, CYS354, TYR355, PHE357
Purple	LEU120, GLY121, CYS123, VAL124, PRO127, VAL142, ASN143, ASN144, HIS145, GLY170, ASP171, PHE172, ASN173, LEU175, THR191, ILE192, GLU193, ALA194, SER195, HIS197, ALA198, LYS199, ARG201, HIS203, CYS204, TYR205, GLU222
Green	ASP243, ILE244, MET245, LYS246, GLY247, ASP248, GLY249, PRO251, GLU252, ILE253, LEU254, VAL321
